# The modification of gelatin films: Based on various cross‐linking mechanism of glutaraldehyde at acidic and alkaline conditions

**DOI:** 10.1002/fsn3.1282

**Published:** 2019-11-19

**Authors:** Junjie Lin, Daodong Pan, Yangying Sun, Changrong Ou, Ying Wang, Jinxuan Cao

**Affiliations:** ^1^ Key Laboratory of Animal Protein Food Processing Technology of Zhejiang Province Ningbo University Ningbo China

**Keywords:** cross‐linking reaction, gelatin films, glutaraldehyde, pH, physical properties

## Abstract

In order to investigate the effect of glutaraldehyde (GTA) on the structure, mechanical properties and thermal stability of gelatin films, gelatin films modified by GTA at various pH (4.5, 6.5, and 11), were prepared. According to FTIR analysis, the reaction mechanism between GTA and gelatin was different at various pH. With the addition of GTA, the intermolecular forces (hydrogen bonds and ionic bonds) and triple helix structure of gelatin film were significantly disrupted. At pH 4.5, gelatin films modified by GTA showed the highest mechanical properties and thermal stability among all films, which tensile strength and residues in TGA up to 16.13 MPa and 15.05%, respectively. Therefore, an optimum pH was around 4.5 in gelatin films cross‐linked by GTA.

## Graphical abstract

The reaction mechanism between glutaraldehyde (GTA) and gelatin was adjusted by pH. pH affected mechanical properties, thermal stability, and triple helix content of the gelatin films with GTA. An optimum pH was around 4.5 in gelatin films cross‐linked by GTA to improve the physical properties of gelatin films.

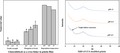

## 1 INTRODUCTION

Recently, biodegradable materials are becoming an important focus in food packaging field due to growing environmental and ecological problems caused by packaging materials of petroleum by‐products. Nature biopolymers including polysaccharides, lipids, and proteins have been demonstrated to be good biodegradable materials, and edible films based on these biopolymers have ability to replace traditional plastic packaging (Nagarajan, Benjakul, Prodpran, & Songtipya, 2015). Among all biopolymers, gelatin, a hydrolysate of collagen, is considered as a promising biopolymer and edible film material which has been widely applied in food, pharmaceutical, and biodegradable packaging materials due to abundance, low cost, nontoxic, and biodegradability (Liu, Antoniou, Li, Ma, & Zhong, 2015; Pena, Caba, Eceiza, Ruseckaite, & Mondragon, 2010). At the process of film making, gelatin undergoes a conformational transition of disorder–order rearrangement and recovers the collagen triple helix structure at the drying process of film (Liu et al., 2015). This property of gelatin contributed to satisfactory mechanical properties of gelatin films. However, the limited mechanical strength is not enough for gelatin films applied in food packaging which is also limited by the high moisture sensitivity and poor thermal stability (Liu et al., 2017).

Cross‐linking response was considered as an effective approach to overcome these problems. Up to now, the cross‐linking way applied in gelatin films mainly included physical, chemical, and enzymic cross‐linking of which chemical ways are the most effective (Benbettaïeb, Gay, Karbowiak, & Debeaufort, 2016). Cross‐linkers involved to chemical cross‐linking contain epoxides, carbodiimides, isocyanates, natural molecules (tannin and ferulic acids), genepin, and aldehyde‐based cross‐linker (formaldehyde, glyoxal, and glyceraldehyde) (Martucci, Accareddu, & Ruseckaite, 2011). For example, glutaraldehyde (GTA) is the most widely used chemical cross‐linker due to low cost, short‐reaction time, and excellent efficiency on the stabilization of collagenous material (Farris, Song, & Huang, 2010). At very low concentration (0.1 wt%–1.5 wt%), GTA has been demonstrated to be nontoxic and is also effective to increase the strength and thermal stability of gelatin films (Bigi, Cojazzi, Panzavolta, Rubini, & Roveri, 2001). It is mainly attributed to Schiff's base between aldehyde and two free amino groups in lysine or hydroxylysine of gelatin (Farris, Schaich, Liu, Piergiovanni, & Yam, 2009).

Recently, cross‐linking reaction of GTA and gelatin was demonstrated to be different at acidic and alkaline conditions where hydroxyl groups of gelatin had a priority to reacted with the aldehyde groups under acidic condition due to the protonation of ε‐amino groups, whereas more free amines are available for Schiff's base at alkaline pH (Farris et al., 2010). Indeed, gelatin can provide multifunctional cross‐linking options for cross‐linkers because of the diversity of side chains groups of gelatin such as amino, carboxyl, and hydroxyl group (Benbettaïeb et al., 2016). These polar groups in gelatin play a crucial role in the stabilization of the triple‐stranded collagen helix, mechanical properties, and thermal stability, especially the hydroxyl of Hyp (Gómez‐Guillén et al., 2002). However, there was no study about the effect of pH on physical–mechanical properties of gelatin films modified by GTA. Although previous study compared the effect of different cross‐linkers on physical–mechanical properties of gelatin films, this research cannot ensure the main factors, because cross‐linker size, polar, and various cross‐linking mechanism had important effect on physical–mechanical properties of gelatin films.

Therefore, in this study, gelatin films with and without GTA were prepared at three different pH values (4.5, 6.5 and 11). The content of helices in films was determined by X‐ray diffraction (XRD). Protein solubility in different solution, mechanical properties, thermal stability, and the morphology water of the films were determined. The objective of the present study was to investigate the effect of the different pH on the structure, mechanical properties, and thermal stability of GTA‐modified gelatin films, which based on different reaction mechanism.

## 2 MATERIALS AND METHODS

### 2.1 Materials and reagents

Gelatin (type B), glutaraldehyde (50 wt% commercialized aqueous regent), and BCA protein concentration assay kit were purchased from Richjoint Chemical Reagent Co.,Ltd. All other reagents were of analytical grade.

### 2.2 Preparation of gelatin films

The gelatin films were prepared according to the method described by Nagarajan et al. (2015) with slight modification. The gelatin solution (6%, *w*/*v*) was prepared by dissolving 6 g gelatin in 100 ml distilled water. The mixture was heated at 60°C for 30 min under continuous stirring until complete dissolution. Glycerol at 25% (*w*/*w*) of protein content was added as plasticizer and stirred at room temperature for 30 min in a magnetic stirrer. The resulting mixture was adjusted to pH 4.5 and 11 with HCl and NaOH, respectively. The pH of solution without HCl and NaOH was detected as 6.5. For GTA‐modified films, 0.2 ml of 50 wt% GTA aqueous solutions was added into gelatin solution with three different pH values, respectively. About 10 ml of film forming solution was cast in polystyrene Petri dishes (9 cm of diameter) and drying at 25°C and 55 ± 5% relative humidity for 48 hr in an environmental chamber (WTB Binder).

The resulted films incorporated with and without GTA at various pH were considered as Gx (*x* = 4.5, 6.5 or 11) and Cx (*x* = 4.5, 6.5 or 11), respectively. Resulting films were peeled off and conditioned at 25°C and 55 ± 5% relative humidity for at least 48 hr in a ventilated climatic chamber prior to testing. For FTIR spectra and TG studies, films were conditioned in a desiccator containing dried silica gel for 2 weeks at room temperature to obtain the most dehydrated films (Liu et al., 2016).

### 2.3 Attenuated total reflectance–Fourier‐transform infrared spectroscopy (ATR‐FTIR)

FTIR spectra of films were recorded using a Bruker Model Equinox 55 FTIR spectrometer (Bruker Co.) equipped with a horizontal ATR Trough plate crystal cell (45° ZnSe; 80 mm long, 10 mm wide and 4 mm thick) (PIKE Technology Inc.) at room temperature as described by Ahmad, Benjakul, Prodpran, and Agustini (2012). Films were placed onto the crystal cell. The cell was clamped into the mount of FTIR spectrometer. The spectra in the range of 400–4000 cm^−1^ with automatic signal gain were collected in 32 scans at a resolution of 4 cm^−1^ and divided by a background spectrum recorded from the clean empty cell at 25°C.

### 2.4 Color

Color of gelatin films was determined using a CIE colorimeter (Hunter associates laboratory, Inc.). The white standard (L* = 93.59, a* = 0.98, b* = 0.35) was used as the background for the measurement.

### 2.5 Protein solubility in various solvents

Solubility of protein in films in various solvents was determined according to Nuanmano, Prodpran, and Benjakul (2015) with some modification. To determine the major associative forces involved in the film matrix, different solvents were used as follow:

S1: 20 mmol/L Tris‐HCl (pH 8.0) containing 1% (w/v) SDS
S2: 20 mmol/L Tris‐HCl (pH 8.0) containing 1% (w/v) SDS and 8 mol/L Urea
S3: 0.6 mol/L NaCl

The samples (0.2 g) were homogenized in various solvents at a speed of 13,000 rpm for 1 min. The resulting homogenates were centrifuged by a microcentrifuge (MIKRO 20, Hettich Zentrifugan) at 7,500 *g* for 30 min. Protein in supernatant (10 ml) was determined by the BCA protein concentration assay kit according to manufacturer' instructions. The solubility was expressed as percentage of the total protein of supernatant in film.

### 2.6 X‐ray diffraction (XRD) analysis

XRD patterns of gelatin films were carried out by means of a PANalytical X’Celerator powder diffractometer using CuKa radiation (40 mA, 40 KV). The relative intensity was evaluated between a range 2θ of 3–60° with a step size of 0.02 and a scanning speed of 4^o^ min^−1^.

### 2.7 Thermogravimetric analysis (TGA)

Thermogravimetric curves were scanned using a thermogravimetric analyser (TGA/1100SF) according to the method of (Ahmad et al., 2012) with minor modification. Dried films were heated in the temperature range of 30–1000°C with a rate of 10°C/min. Nitrogen was used as the purge gas at a flow rate of 20 ml/min.

### 2.8 Mechanical properties

Elastic modulus (EM), tensile strength (TS), and elongation at break (EAB) were determined using ASTM 2001 method as described by Hoque, Benjakul, and Prodpran (2010) using a microcomputer control electronic universal testing machine (CMT6202, MTS systems CO., LTD.). Ten samples (2 × 5 cm^2^) with initial grip length of 3 cm were used for testing. Samples were clamped and deformed under tensile loading using a 100 N load cell with crosshead speed of 30 mm/min until samples were broken. The maximum load and the final extension at break were used for calculation of TS and EAB, respectively. EM was calculated as the initial slope of the linear portion of stress–strain curve. Fracture surface of the broken gelatin films was defined as fractography.

### 2.9 Statistical analysis

All experiments were performed with different three films. Completely randomized design was used. Data were subjected to analysis of variance (ANOVA). Mean comparisons were carried out by Duncan's multiple range test in SAS 8.0 (SAS Institute Inc.). *p* < .05 was considered as significant.

## 3 RESULTS AND DISCUSSION

### 3.1 ATR–FTIR analysis

**Figure 1:**
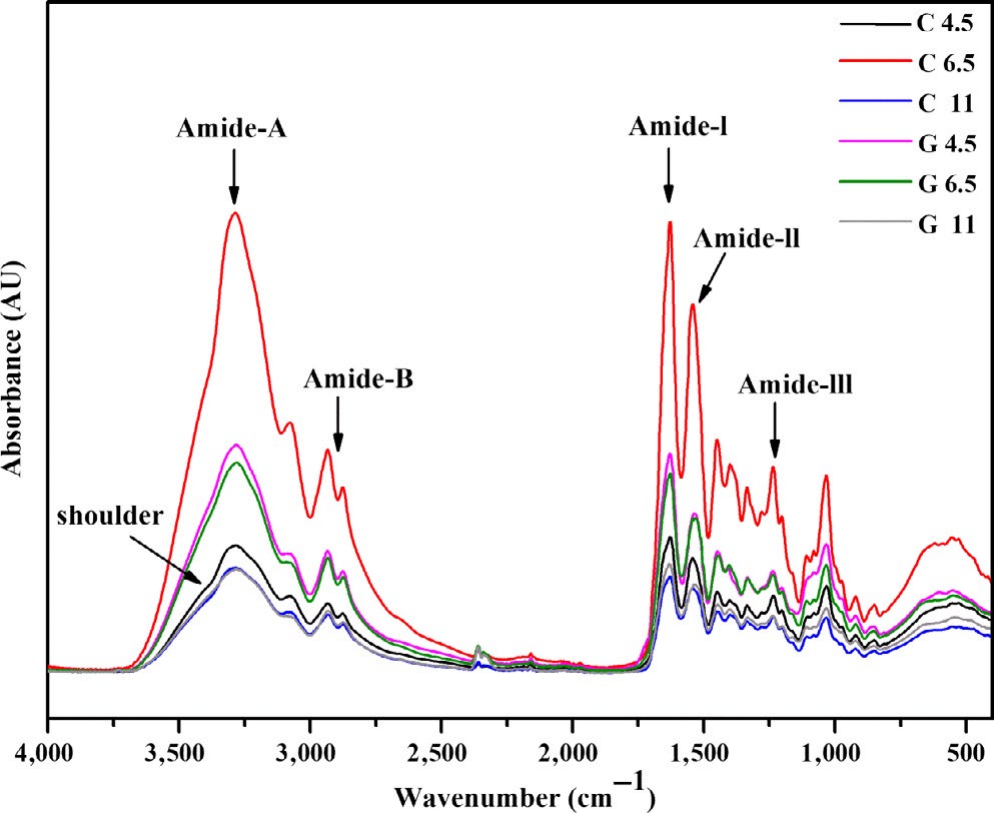
Fourier transform infrared spectra of gelatin films incorporated with and without GTA at various pH

FTIR spectra of gelatin films added with and without GTA at selected pH were displayed in Figure 1. All gelatin films showed major peaks in amide region which showed slight difference in the spectra. Amide‐A band, arising from N‐H stretching vibration coupled with ‐OH groups via hydrogen bonding (Ahmad et al., 2012), was observed at wave numbers of 3,283, 3,285, 3,286, 3,282, 3,280, and 3,282 cm^−1^ for the C 4.5, C 6.5, C 11, G 4.5, G 6.5, and G 11 film, respectively. The shift of the amide‐A band of films toward lower wave numbers was observed when GTA was incorporated. It was attributed to the scission of hydrogen bonds necessary to maintain the helical structure of collagen (Staroszczyk, Pielichowska, Sztuka, Stangret, & Kołodziejska, 2012). The results suggested the difference in the degree of destruction of hydroxyl at various pH. These results were in agreement with protein solubility at S1 (Table 1). Moreover, Amide‐II peak, representing bending vibration of N–H groups and stretching vibrations of C–N groups (Tongnuanchan, Benjakul, Prodpran, & Nilsuwan, 2015), was detected at the wavenumbers of 1,543, 1,543, 1,543, 1,537, 1,534, and 1,537 cm^−1^ for C 4.5, C 6.5, C 11, G 4.5, G 6.5, and G 11 films. A shift of amide‐II to lower wavenumber with the addition of GTA suggested the existent of intermolecular interaction, which was consistent with gelatin films cross‐linked by transglutaminase (Staroszczyk et al., 2012).

**Table 1:** Protein solubility in various solvents and b* value of gelatin films incorporated with and without GTA at various pH

Samples	Protein solubility (%)			b* value
	S1	S2	S3	
C 4.5	11.14 ± 0.39^a^	11.62 ± 1.13^a^	11.01 ± 0.56^a^	−1.57 ± 0.08^a^
C 6.5	11.94 ± 0.2^b^	12.69 ± 0.87^a^	12.47 ± 0.01^b^	−1.48 ± 0.14^a^
C 11	7.73 ± 0.31^c^	9.43 ± 0.24^b^	5.49 ± 0.24^c^	0.06 ± 0.3^b^
G 4.5	3.22 ± 0.13^d^	4.03 ± 0.42^c^	1.07 ± 0.03^d^	35.48 ± 0.74^c^
G 6.5	0.76 ± 0.21^e^	0.96 ± 0.03^d^	1.08 ± 0.07^d^	40.82 ± 0.85^d^
G 11	0.57 ± 0.03^e^	1.52 ± 0.43^d^	1.19 ± 0.02^d^	46.83 ± 0.75^e^

Note

Values are given as mean ± *SD* (*n* = 3).

Different letters in the same column indicate significant differences (*p* < .05).

Finally, it was clear that the addition of GTA caused the lower intensity of “shoulder‐like” peak at pH 4.5 and 6.5 than 11, which centered at around 3,350 cm^−1^ and corresponded to the free ‐OH stretching band. This was consisted with Farris et al. (2010) who found GTA could react with ‐OH and ‐NH_2_ of gelatin via Schiff base and hemiacetal reaction at acidic and alkaline condition, respectively. Therefore, these findings indicated that the reaction of GTA and gelatin is dependent on pH.

### 3.2 Color

Color is an important attribute for gelatin films application and determined by L*, a* and b* parameters. Etxabide, Urdanpilleta, Guerrero, and Caba (2015) reported that only b* value was significantly affected by Maillard reaction, and thus a* value and L* value did not present. b* value of gelatin films added with or without GTA at selected pH was shown in Table 1. At the same pH, the incorporation of GTA remarkably increased b* value of gelatin films *(p* < .05). Among all pH, gelatin films showed highest b* value at pH 11 (*p* < .05). The result suggested that alkaline condition is beneficial for yellowish pigment formation in the solution. It might be due to the more ‐NH_2_ at alkaline condition. Hoque et al. (2010) found that the free amines of lysine of gelatin could react with glycerin molecules, and thus increasing b* value of gelatin films. Additionally, b* value of gelatin films significantly increased with the addition of GTA. Pervious study reported that Schiff's bases between amide groups of gelatin and GTA contribute to the increased b* value (Martucci et al., 2011). When GTA was incorporated, the different b* value of gelatin films suggested the different level of Schiff's bases at above pH. This was consistent with Farris et al. (2009) who reported that amino groups of gelatin were protonated at pH 4.5, restricting Schiff's bases. Hence, the difference of b* value of GTA‐modified gelatin films at various pH might be associated with the interaction mechanism of GTA and gelatin.

### 3.3 Protein solubility in various solvents

The formation of gelatin film is believed to the renaturation of a triple helical structure of protein molecules which was mainly stabilized by ionic, hydrogen, hydrophobic, and covalent bonds. Therefore, the solubility of the films in the various solutions revealed the associative force involved in the formation of film, containing S1 (the disruption of hydrogen bonds), S2 (disruption of hydrophobic interactions), and S3 (disruption of ionic bonds) (Nuanmano et al., 2015). Protein solubility in these solutions of gelatin films incorporated with and without GTA at various pH was shown in Table 1. The higher protein solubility of gelatin films at S2 than S1 was observed, which suggested that hydrophobic interactions were involved in the formation of gelatin films. In the absence GTA, the films at pH 6.5 showed higher solubility than 4.5 and 11. It might be due to electrostatic repulsion of negative or positive charge at pH 4.5 and 11, leaving the number of junction zones decreases by hydrogen bonds. The results were consistent with Hamaguchi, Wuyin, and Tanaka (2007).

**Figure 2:**
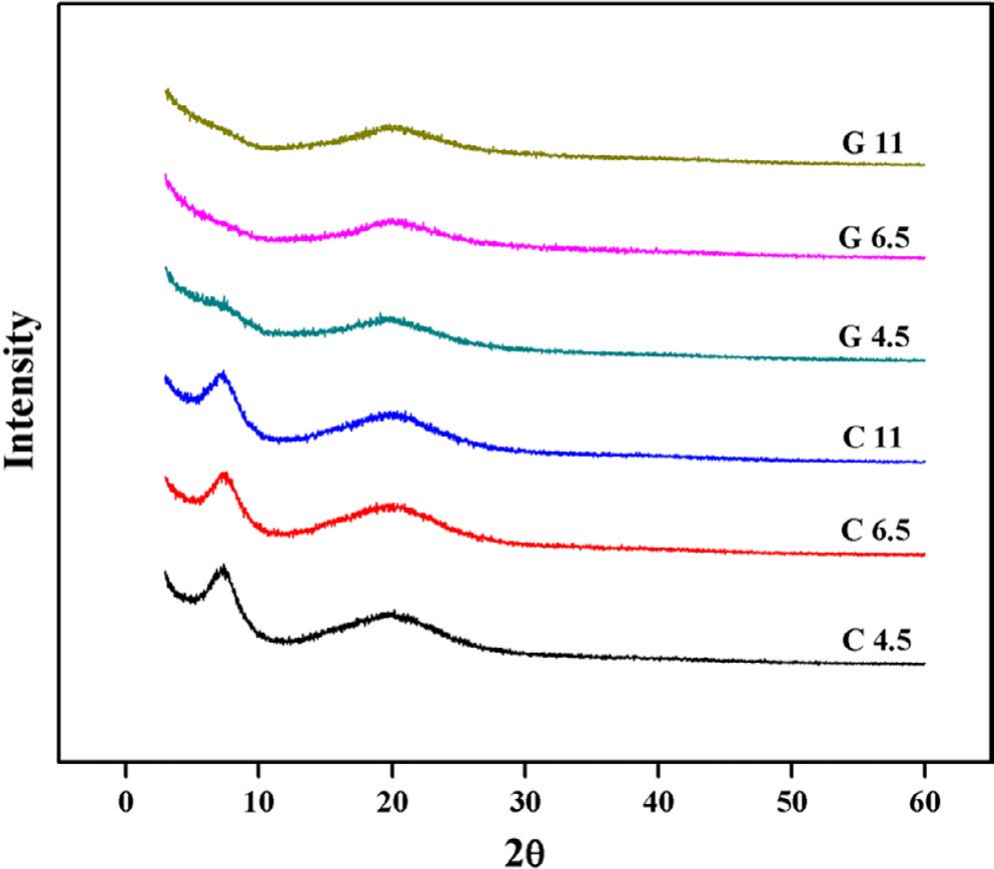
XRD patterns of gelatin films incorporated with and without GTA at various pH

When GTA was added, especially at pH 6.5 and 11. These decreases in protein solubility might be attributed to the formation of covalent bonds. Guo, Ge, Li, Mu, and Li (2014) reported that the cross‐linking reaction between gelatin and periodate oxidation of xanthan gum diminishes interactions of gelatin chains such as hydrogen bonding, electrostatic forces, and van der Waals forces. The higher solubility at G4.5 for GTA films might be related to the reaction mechanism between gelatin and GTA at various pH. because of the protonation of amino groups at acidic condition, GTA interacts with ‐OH of gelatin at pH 4.5 (Farris et al., 2010). It is well‐known that hemiacetals reaction between aldehyde and hydroxyl groups could produce a new hydroxyl, and thus we speculate that the new hydroxyl can reform hydrogen bond with other polarity groups of gelatin, resulting in the lower destroy of hydrogen bonds at pH 4.5 than 11. Additionally, since hydroxyl groups of the hemiacetal reaction extended out from the triple helix (Farris et al., 2009), and thus inferring this hydroxyl groups is not involved in the formation of hydrogen bond. This result was in agreement of the degree of damage of triple helix, as observed in Figure 2.

### 3.4 XRD analysis

XRD patterns of gelatin films with or without GTA at different pH were shown in Figure 2. It exhibited a sharp peak and a broad peak at 2θ around 7° and 20° and these peaks occurred for selected gelatin films did not show displacements. The intensity of the peak at 2θ 7° was considerably reduced with GTA added, especially at pH 11 where the peak at 2θ 7° almost disappeared. The peak at 2θ around 7° represented the diameter of triple helical structure, and its intensity represented the content of triple helix structure (Cheng et al., 2019). These results indicated that the diameter of the triple helix structure did not be affected by GTA and pH, but the content of triple helix structure was markedly disrupted by the addition of GTA. The similar results were also found by Liu et al. (2016) who prepared gelatin films cross‐linked by transglutaminase, which is attributed to the formation of covalent bond preventing the renaturation of triple helix structure.

Indeed, triple helical structure of collagen was mainly stabilized by hydrogen bonds (Pena et al., 2010). The hydrogen bonds of gelatin films were markedly destroyed by the addition of GTA (Table 1), and thus resulting in the lower content of triple helix structure for GTA films. Additionally, Farris et al. (2009) reported that hydroxyl groups of the hemiacetal reaction extended out from the triple helix. This might be another reason why G4.5 had the highest content of triple helical structure among GTA films.

### 3.5 Thermogravimetric analysis

**Figure 3:**
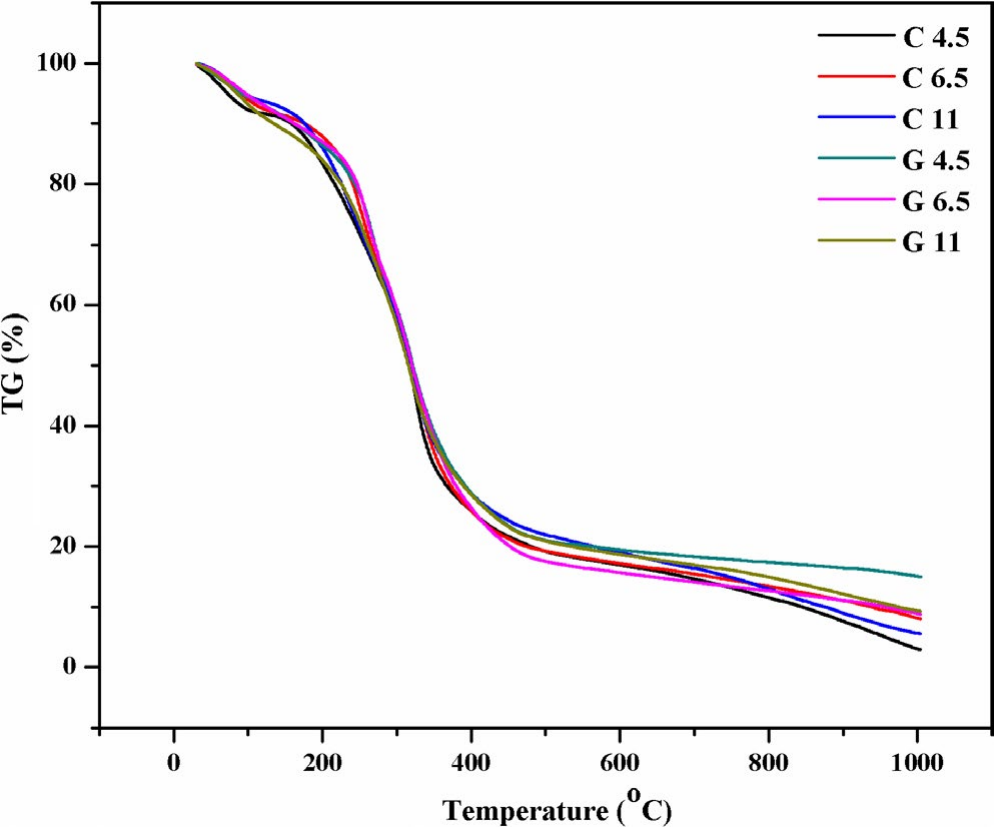
Thermogravimetric curves of gelatin films incorporated with and without GTA at various pH

Thermogravimetric analysis (TGA) was performed to evaluate thermal stability of gelatin films incorporated with and without GTA, displayed in Figure 3. The weight loss (∆*w*) and residue of all film samples were presented in Table 2. All films showed three main weight loss stages. The first stage (∆*w*_1_) was a 5.41%–10.69% weight loss for all films, represent water absorbed in the film (Inamura et al., 2013; Tongnuanchan et al., 2015). The second stage (∆*w*_2_) showed 22.59%–32.14% weight loss, which is associated with the loss of glycerol and the degradation of low molecular weight protein fraction, as well as structural bound water. A 47.5%–61.2% weight loss was observed at the third stage (∆*w*_3_) which depends on the degradation of larger size or higher interacted protein fractions (Tongnuanchan et al., 2015).

**Table 2:** Weight loss (∆*w*, %) and residual mass (%) of films incorporated with and without GTA at various pH

Films samples	∆*W*1	∆*W*2	∆*W*3	Residue (%)
C 4.5	7.84	27.6	61.2	3.06
C 6.5	8.1	26.9	56.76	8.15
C 11	6.25	29.5	58.85	5.67
G 4.5	5.41	32.4	47.5	15.05
G 6.5	7.69	27.8	55.6	8.9
G 11	10.69	22.6	57.32	9.39

It was noted that GTA reduced ∆*w*_1_, but increased ∆*w*_2_ at the pH 4.5 and 6.5 which was contrary to pH 11. This might be related to the different cross‐linking extent caused by various cross‐linking mechanism at various pH with the addition of GTA. Martucci et al. (2011) reported that the low cross‐linking extent achieved by GTA let the plasticizer to expand the network which displayed a strong plasticizing effect and water‐bonding capacity. Indeed, at pH 11, glycerol can penetrate into the GTA–gelatin network. The result was demonstrated by DTG (be not shown) which depicted the two maximum degradation peaks almost merged into only one maximum degradation peak for G11 groups. This also explained the highest elongation at break of G11 among GTA films. Moreover, ∆*w*_3_ of films decreased as the addition of GTA, especially for G4.5. It indicated that the addition of GTA could improve the thermal stability of gelatin films, in agreement with the results observed by Bigi et al. (2001). The results were also consistent with residual mass at 1,000°C.

### 3.6 Mechanical properties

**Figure 4:**
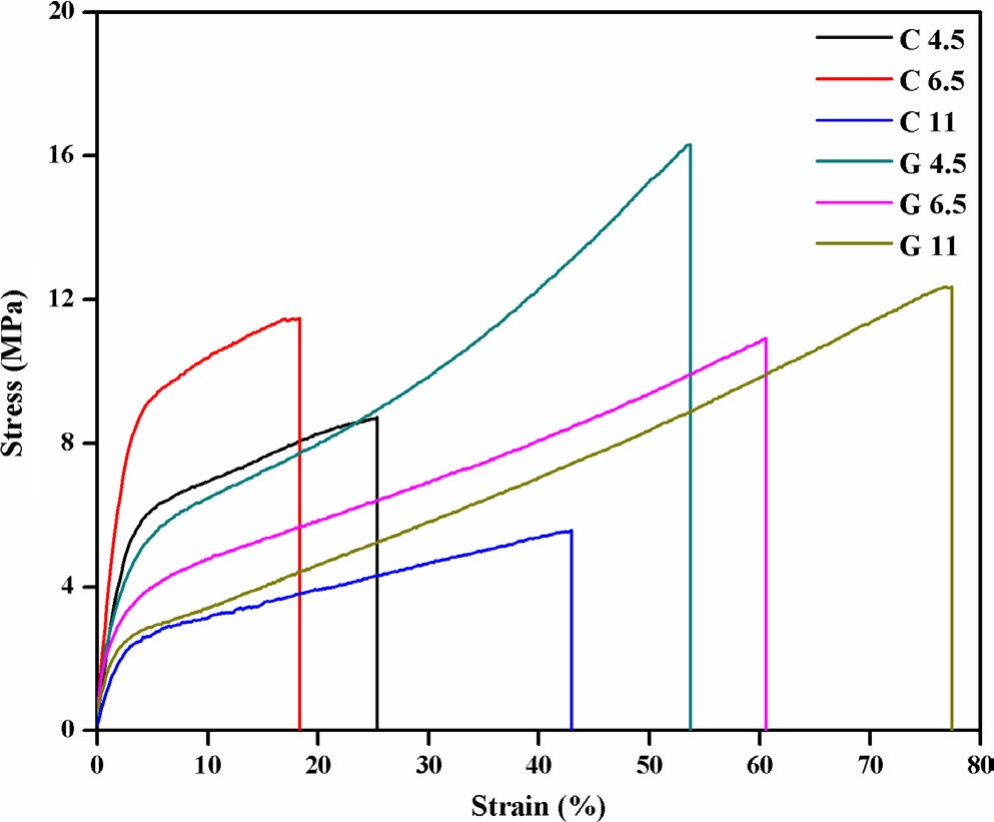
Typical stress–strain curves of gelatin films incorporated with and without GTA at various pH

Representative stress–strain curves from tensile tests of gelatin films were shown in Figure 4, and the related parameters were summarized in Table 3. C6.5 showed highest tensile strength (TS), but lowest elongation at break (EAB) among control films. The similar results were reported by Gioffrè, Torricelli, Panzavolta, Rubini, and Bigi (2012), which might be due to that a net positive and negative charge caused electrostatic repulsion at pH 4.5 and 11, resulting in the increase in free volume and flexibility of gelatin films.

**Table 3:** Elastic modulus (E), tensile strength (TS) and elongation at break (EAB) of gelatin films incorporated with and without GTA at various pH

Film samples	E (MPa)	TS (MPa)	EAB (%)
C 4.5	243.41 ± 9.37^a^	8.44 ± 0.24^ab^	25.66 ± 3.7^a^
C 6.5	370.51 ± 13.97^b^	11.98 ± 0.44^c^	18.11 ± 1.84^a^
C 11	111.64 ± 0.36^c^	6.22 ± 1.31^d^	42.43 ± 1.56^b^
G 4.5	179.58 ± 9.70^d^	16.13 ± 3.7^d^	53.34 ± 3.11^c^
G 6.5	164.91 ± 4.32^e^	10.84 ± 0.69^ac^	62.92 ± 2.12^d^
G 11	76.67 ± 3.26^f^	12.42 ± 0.32^c^	78.96 ± 10.12^e^

Note

Values are given as mean ± *SD* (*n* = 3).

Different letters in the same column indicate significant differences (*p* < .05).

At the same pH, the addition of GTA caused the increase in both TS and EAB, but decrease in EM reduced. G4.5 showed highest TS and EM but lowest EAB among GTA films. It was contrary to Ge et al. (2015) who reported that the increase in EAB of protein films due to cross‐linking was often accompanied by reduced film TS. From these results, EAB and EM were inversely related, which meant that films with higher elongation values were more prone to plastic deformation. The yield point corresponds to the onset of plastic deformation of the polymer and is mainly related to cooperative movement of polymer chain segment (Stachurski, 2003). The addition of GTA prevented renaturation of triple helical structures of gelatin and caused the increase in random‐strand molecular structure content which made the cooperative movement of polymer chain segment easier. The random‐strand molecular had plasticizing effect on gelatin (Guo et al., 2014). Therefore, the addition of GTA caused EAB increased, but EM reduced. Furthermore, because water molecules could play the role of plasticizer in the gelatin films (Vieira, Silva, Santos, & Beppu, 2011), the high water content (Table 2) of G11 is also the main reason leading to the highest EAB of G11.

On the other hand, the improvement of TS caused by GTA mainly happened after yield point which was related to engagement of entanglements between the deforming gelatin chains (Stachurski, 2003). Deformation with an increasing drawing stress at this process is mainly due to the formation of molecular orientation (Stachurski, 2003). Furthermore, the results also related to the formation of compact and denser network structure through cross‐linking reaction. The same behavior was observed in gelatin films modified by transglutaminase (Liu et al., 2016).

## 4 CONCLUSIONS

In this work, we found that pH greatly affects mechanical properties and thermal stability of gelatin films, which display decreasing values of elastic modulus and reduced residues at pH 4.5 and 11 compared with 6.5. With the addition of GTA, the intermolecular forces (hydrogen bonds and ionic bonds) and triple helix structure of gelatin film were significant disrupted. Furthermore, mechanical properties and thermal stability of gelatin film were enhanced by GTA, especially at pH 4.5. Therefore, an optimum pH was around 4.5 in gelatin films cross‐linked by GTA.

## INFORMED CONSENT

Written informed consent was obtained from all study participants.

## CONFLICT OF INTEREST

The authors declare that they do not have any conflict of interest.

## ETHICAL APPROVAL

This study does not involve any human or animal testing.
